# Evaluating the affordability of asthma, chronic obstructive pulmonary disease, and cystic fibrosis medicines in a middle-income country

**DOI:** 10.1186/s12890-023-02737-5

**Published:** 2023-11-04

**Authors:** Mahdieh Fathi, Najmeh Moradi, Nazila Yousefi, Farzad Peiravian, Nikta Shobeiri

**Affiliations:** 1 Department of Pharmacoeconomics and Pharma Management, School of Pharmacy, Shahid Beheshti University of Medical Sciences, Tehran, Iran; 2 Population Health Sciences Institute, Newcastle University, Newcastle upon Tyne, UK; 3 Department of Pharmaceutical Control, School of Pharmacy, Mashhad University of Medical Sciences, Mashhad, Iran

**Keywords:** Chronic respiratory disease, Affordability, Medication cost, Developing countries, Out-of-pocket

## Abstract

**Background:**

A heavy financial burden is imposed on patients suffering from chronic diseases due to medicine out-of-pocket payments.

**Objectives:**

This study focuses on assessing the affordability of medications used for chronic respiratory diseases (CRDs) such as asthma, chronic obstructive pulmonary disease (COPD), and cystic fibrosis (CF) in Iran, specifically on the category R medicines listed in the 2017 Iran drug list (IDL) that are used for the treatment of these diseases, based on the anatomical therapeutic chemical (ATC) drug code.

**Methods:**

The affordability of medicines in mono and combination therapy approaches was assessed in CRDs using the World Health Organization/Health Action International (WHO/HAI) methodology. Accordingly, if out-of-pocket payment for 30-days of pharmacotherapy exceeds one day for the lowest-paid unskilled government worker (LPGW), it’s considered non-affordable.

**Results:**

Based on the monotherapy approach, our finding demonstrates that all generic medicines of category R were affordable. However, branded drugs such as Symbicort®, Pulmicort Respules®, Flusalmex®, Seretide®, Fluticort Plus®, Seroflo®, and Salmeflo® cost between 1.2 and 2.5 days’ wage of LPGW and considered unaffordable despite 70% insurance coverage. Moreover, based on the affordability ratio in the combination therapy approach, all medicines used in asthma, COPD, and CF patients with mild respiratory problems are affordable except omalizumab (inj), which is non-affordable due to its high price and no insurance coverage.

**Conclusion:**

Results showed that the existing insurance coverage does not protect households from hardship, so more considerations are needed such as different insurance schedules and patient support programs.

## Introduction

Chronic respiratory diseases (CRDs) are chronic diseases of the airways and other parts of the lung. Asthma and chronic obstructive pulmonary disease (COPD) are the most common CRDs affecting patients, their families, and society. CRDs account for about 4.7% of global disability-adjusted life years (DALYs) [1]. The global burden of disease (GBD) study (2017) indicates that DALYs due to CRDs ranged from 97.2 to 112.3 million a year from 1990 to 2017 [2]. In Iran, the burden of CRDs in both sexes was estimated at about 872.1 DALYs per 100,000 population in 2003 [3].

According to the world health organization (WHO) key facts, 262 million people worldwide have asthma [4]. According to the estimate projected by the institute for health metrics and evaluation (IHME), the burden of asthma disease is about 21.6 million DALYs. Moreover, it is revealed that countries with low and middle socio-demographic index (SDI) have experienced higher asthma deaths compared to countries with high SDI [5]. In Iran, a meta-analysis of population–based studies on the prevalence of asthma from 1990 to 2015 showed that the pooled prevalence of asthma is 7.95% [6]. The prevalence of asthma in Iran in adults aged 20 to 44 years old in a cross-sectional survey in 2015–2016 was estimated at about 8.9% [7]. Asthma puts a significant financial burden on the health system and reduces productivity in the workplace. Of all the total national DALYs, 4.36% is related to CRDs and 0.77% is dedicated to asthma [3].

With the dramatic increase in the prevalence and mortality of COPD over the past two decades, this disease has become the third main reason of death all around the world, with 3.23 million deaths in 2019, and accounts for more than 80% of deaths taking place in low-and middle-income countries [8]. The evidence of GBD in 2017 showed that COPD was the seventh major cause of years of life lost (YLL) [9].

In Iran, COPD was ranked 9th among the major causes of death in 2009, while in 2019 it occupied the 7th position, with an increase of 41.9% in the number of cases reported [10]. Due to the lack of accurate data on the prevalence of COPD in Iran, different prevalence rates have been reported from 1.68 to 10% [11, 12].

Cystic fibrosis (CF) is a fatal chronic genetic disease, which principally affects the respiratory and gastrointestinal systems, leading to progressive disability [13]. About 70,000 to 100,000 people around the world have been affected by CF [14]. The WHO has reported that the incidence of CF in European newborns is 1 in 2000–3000. According to the same report, 1 in 3500 newborns is affected by CF in North America; no accurate data is available in Africa, and in the Middle East it differs from 1 to 2560 to 1 in 15,876 newborns [15]. Various incidence rates of CF have been reported in Iran. Some studies have reported no confirmed case of CF in southern Iran [16], while, according to another report, in the northwest of Iran 7.98 out of 100,000 people suffered CF during a four-year period ranging from 2004 to 2008 [17]. Considering the high burden of respiratory diseases on governments and individuals including disability, premature deaths, loss in productivity and treatment expenses, medicine affordability in the disease treatments is a crucial issue, particularly in low-resource settings. Due to the lack of comprehensive health insurance coverage and the unavailability of cheap or free medicines through the public sector in developing countries, people have to pay out-of-pocket to procure the medicines, therefore a large part of the total health care expenses is related to the medicine cost [18]. At the same time, medicine prices are high and people may go into debt or stop treatment. Consequently, the WHO has set affordable prices as a leading factor in medicine accessibility, along with rational selection and use, sustainable financing, and reliable health and supply systems [19].

The present research sought to explore the affordability of Asthma, COPD, and CF in Iran. The results of affordability studies contribute to developing and improving financial protection policies across health systems.

### Rationale of the study

There is a rise in healthcare expenses among Iranians. Total expenditure in the Iranians health sector increased from $24.3 billion in 2008 to $96 billion by 2017comprises about 7% of the gross domestic product (GDP) expenditure [20]. This condition leads to increasing out-of-pocket (OOP) payments and catastrophic health expenditures (CHEs); therefore, a large portion of society is unable to afford healthcare expenses which put their health at risk [21]. More than 50% of the health expenses are being paid by people in Iran [22]. The share of medicine in those OOP payments is significant [23]. Therefore, the WHO and other organizations have been working towards increasing affordability of healthcare, particularly medicines, for various diseases [18, 19, 24]. The WHO has set affordable prices as a leading factor in medicine accessibility, along with rational selection and use, sustainable financing, and reliable health and supply systems [19]. As such, To the best of the researcher’s knowledge, the current research is the first comprehensive attempt to examine CRDs medicine affordability in mono and combination therapy approaches.

## Methods

This is a cross-sectional study that assessed the affordability of mono and combination medication therapy in asthma, COPD, and CF diseases in Iran’s health system in 2017 by adopting the WHO/HAI methodology [25]. Accordingly, the affordability of medications is defined as the number of days’ wages the lowest-paid unskilled government worker has to spend on a particular medicine for a course of treatment [26]. If the cost of payment exceeds one day’s salary, it is considered unaffordable [27]. While for acute illnesses the cost is calculated for a period of treatment, for chronic diseases it is estimated for a period of 30 days [28]. There is no ethical approval required for this study.

The steps of affordability assessment are outlined here:

A) All asthma, COPD, and CF medications listed in the 2017 Iran drug list (IDL) (a generic-based list of medicines maintained by the Iran Drug Selection Committee, serving as the main base for pharmaceutical activities and prescriptions in Iran) were elicited with various dosage forms and strengths based on the anatomical therapeutic chemical (ATC) drug code (category R medicines).
B) The treatment schedules for asthma, COPD, and CF were defined by using three main international guidelines including “Applied Therapeutics: The Clinical Use of Drugs” [29], the Global Initiative for Chronic Obstructive Lung Disease (GOLD) guideline for asthma management [30], and Pulmonary Clinical Practice Guidelines supported by the Cystic Fibrosis Foundation [31]. Afterward, in order to estimate the price of the daily dose of each medicine, the defined daily dose (DDD) (i.e., the average daily maintenance dose for a drug in its main indication) was utilized.
C) There are three main insurance funds provide basic health insurance coverage in Iran. These funds include: Iran Health Insurance Organization (IHIO), Social Security Organization (SSO), and Armed Forces Medical Services Insurance Organization [4]. Currently, 45,700,000 people in Iran are covered by Iran health insurance, which covers more than 50% of the population [4]. As the largest insurance fund in Iran, IHIO was selected for collecting data on insurance coverage of CRDs’ medications. The price data was extracted from Iran Food and Drug Administration. The data on the lowest-paid unskilled government worker (LPGW) was gleaned from the Ministry of Cooperatives Labor and Social Welfare (370,000 Iranian Rials per day in 2017).
D) Finally, the affordability ratio was estimated through dividing patients’ OOP by the minimum daily wage for each treatment schedule in combination therapy. If this ratio was more than 1, the patient’s OOP for medication therapy would exceed one day of LPGW and the treatment was regarded as non-affordable; otherwise, it was classified under the affordable category.

## Results

The affordability of CRD medicines was investigated and presented for each disease using the LPGW method. The results are as follows:

### Monotherapy treatment approach

**Fig. 1:**
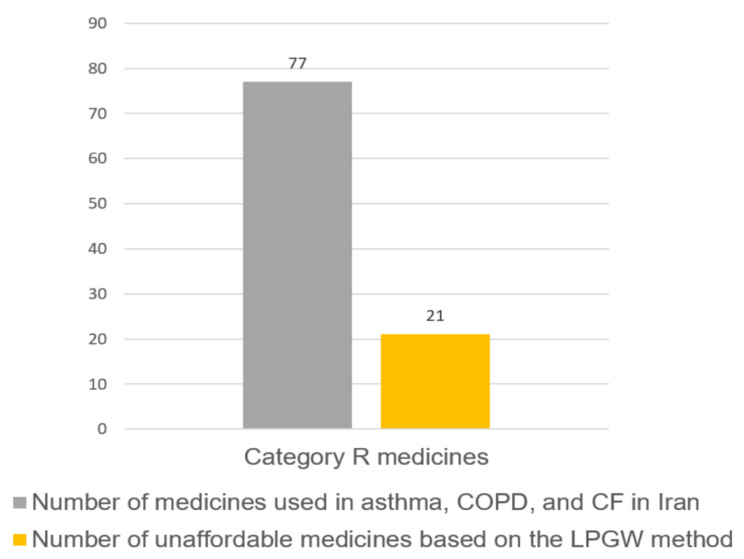
The affordability of medicines used in asthma, COPD, and CF, based on the monotherapy treatment approach

In the monotherapy approach, the price of each drug for a course of treatment was assessed after deducting the percentage of insurance coverage and the affordability of each drug was determined according to the minimum daily wage of the LPGW. For this purpose, the affordability of all generic and branded medicines used in asthma, COPD, and CF from R category that are available in Iran were evaluated separately. Figure 1 indicates that, out of the 77 medicines used in asthma, COPD, and CF, 21 are unaffordable.

Based on the results obtained through the monotherapy approach, all generic medicines were affordable, which can be attributed to the low prices and insurance coverage of generic medicines. Interestingly, in some cases, branded medicines included budesonide/formoterol (Symbicort®m) budesonide (Pulmicort Respules®), salmeterol/fluticasone (Flusalmex®), (Seretide®) (Fluticort Plus®), (Seroflo®) and (Salmeflo®) costs between 1.2 and 2.5 days’ wage of LPGW and considered unaffordable despite 70% insurance coverage. Table 1 demonstrated the unaffordable drugs with characteristics such as their brand name, dosage form and strength, insurance coverage and the number of days’ wage of LPGW needed to procure the medicine.

**Table 1:** Unaffordable medicines used in asthma, COPD and CF

	Generic name	Brand name	Dosage form	Dose	Insurance coverage percentage	Days wage of LPGW
1	Budesonide	Pulmicort respules®	Nebulisation	0.5 mg/2ml	70	2.2
2	Budesonide	Pulmicort respules®	Nebulisation	1 mg/2ml	70	1.7
3	Budesonide + formoterol	Budeform®	Inhaler	320mcg/9 mcg/dose	0	3
4	Budesonide + formoterol	Symbicort®	Inhaler	320mcg/9 mcg/dose	70	1.2
5	Ipratropium + salbutamol	Duolin®	Nebulisation	100 mcg / 20 mcg	0	13.8
6	Mometasone furoate	Asmanex	Powder, inhalation	200 mcg	0	1.9
7	Mometasone furoate	Asmanex	Powder, inhalation	400 mcg	0	1.9
8	Mometasone + formoterol	Zenhale	Aerosol, inhalation	200mcg/5mcg	0	2
9	Montelukast	Singulair	Granule	4 mg	0	3.2
10	Salmeterol + fluticasone	Seretide	Aerosol, metered	25mcg/250mcg	70	2.5
11	Salmeterol + fluticasone	Fluticort plus	Aerosol, metered	25mcg/250mcg	70	1.5
12	Salmeterol + fluticasone	Salmeflo	Inhaler	25mcg/250mcg	70	2.4
13	Salmeterol + fluticasone	Seroflo	Aerosol, metered	25mcg/125mcg	70	1.6
14	Salmeterol + fluticasone	Flusalmex	Inhaler	25mcg/50mcg	70	2
15	Salmeterol + fluticasone	Salmeflo	Aerosol, metered	25mcg/125mcg	70	1.4
16	Salmeterol + fluticasone	Seretide	Aerosol, metered	25mcg/125mcg	70	2.5
17	Salmeterol + fluticasone	Seretide discus	Disc	50mcg/500mcg	0	1.9
18	Salmeterol + fluticasone	Seretide discus	Disc	50mcg/250mcg	0	2.2
19	Salmeterol + fluticasone	Seretide discus	Disc	50mcg/100mcg	0	1.3
20	Omalizumab	Xolair	Injection, powder	150 mg	0	20.8
21	Sodium chloride		Nebulisation	7%	0	17

### Combination therapy approach

#### Affordability of asthma medication therapy

For determining treatment schedules in asthma management, a stepwise approach was used to adjust the treatment of asthma according to the guideline. Asthma treatment is categorized into 6 steps based on the severity of the disease [27]. The stepwise approach to asthma treatment is shown in Table. The cheapest medications that are used as the preferred therapy at each step are listed in this table. According to the guideline, omalizumab (inj) is added for patients who have allergies at steps 5 and 6.

**Table 2:** The affordability of asthma, COPD and CF

	Therapeutic category	Drug class	Drug name	Affordability ratio
Asthma	Step^*^ 1	SABA PRN	Salbutamol (100mcg/dose)	0.11
	Step 2	Low dose ICS	Fluticasone (125 mcg/dose)	0.2
	Step 3	Medium dose ICS	Budesonide (200 mcg/dose)	0.88
	Step 4	Medium dose ICS + LABA	Budesonide (200 mcg/dose) + Formoterol (12 mcg)	0.15
	Step 5	High dose ICS + LABA AND Omalizumab (For allergic patients)	Fluticasone (250 mcg/dose) + Formoterol (12 mcg) AND Omalizumab (150 mg)	0.2 31.05
	Step 6	High dose ICS + LABA + OCS AND Omalizumab (For allergic patients)	Fluticasone (250 mcg/dose) + Formoterol (12 mcg) + Prednisolone (5 mg) AND Omalizumab (150 mg)	0.21 31.10
COPD	Group^**^ A	SABA	Salbutamol (100mcg/dose)	0.11
	Group B	LABA	Formoterol (12 mcg)	0.07
	Group C	LAMA	Tiotropium (18 mcg/capsule)	0.25
	Group D	LAMA + LABA	Tiotropium (18 mcg/capsule) + Formoterol (12 mcg)	0.32
CF	NA	Hypertonic Saline + SABA AND ICS (For CF patients who have definite signs of asthma)	Sodium chloride solution respiratory 7% (4ml/dose) + Salbutamol (100mcg/dose) AND Fluticasone (250mcg/dose)	0.11 0.24

**Note**: SABA is short-acting beta2-agonist; LABA is long-acting beta2-agonist; ICS is inhaled corticosteroids; OCS is oral corticosteroids; PRN is pro re nata; LAMA is long-acting muscarinic antagonist; NA is not applicable; COPD is chronic obstructive pulmonary disease; CF is cystic fibrosis

^*^ For determining treatment schedules in asthma management, a stepwise approach was used to adjust the treatment of asthma according to the guideline.

^**^COPD patients were divided into 4 groups based on the level of symptoms and the risk of exacerbations following the guideline.

Results show the affordability ratio of all steps is less than 1, indicating the affordability of asthma treatment in Iran. However, if omalizumab (inj) is added to the therapeutic regimen of patients who have allergies in steps 5 and 6, the affordability ratio will increase dramatically due to the high price of omalizumb (inj). Hence, omalizumab (inj), which is not covered by insurance, is considered unaffordable in asthma treatment.

#### Affordability of COPD medication therapy

Following the GOLD guideline, COPD patients were divided into 4 groups based on the level of symptoms and the risk of exacerbations (number of moderate or severe exacerbations in the past year) [30–32]. The suggested initial treatment for COPD patients is shown in Table. It is recommended that patients in groups A, B, and C receive bronchodilators as initial treatment. As patients in group D are symptomatic and at risk of exacerbations, the initial treatment for these patients depends on the severity of symptoms and even the count of the blood eosinophil [29]. Noteworthy, we have listed the cheapest medications used in each group.

According to the results displayed in Table 2, the affordability ratio of medicines used in COPD is far less than 1, demonstrating the affordability of COPD treatment in Iran.

#### Affordability of CF medication therapy

Patients with CF are difficult to treat because the signs and symptoms as well as the severity of disease symptoms vary from person to person. Some people may experience serious complications, while others may only suffer from respiratory problems without experiencing other complications [33]. Thus, complex therapeutic approaches are required to improve the quality of life of patients with CF [34].

The Cystic Fibrosis Foundation (CFF) published guidelines on the treatment of CF that contains individualized, pharmacological, and non-pharmacological treatment [35]. Combination therapy for the treatment of CF is not included in this study because a combination of antibiotics, gene modulators, inhaled corticosteroids, etc. is needed for the treatment of this disease, which is outside the scope of the current study. For this purpose, a dedicated study is needed to evaluate the affordability of CF medicines in patients with serious complications. According to the scope of the present study, the affordability of medicines used in patients with mild respiratory problems was explored using CFF guidelines.

Hypertonic saline is an expensive drug in Iran, which is not covered by insurance. To support CF patients, however, this drug is provided free of charge by the government for patients who registered in CF society. As reported in Table 2, the affordability ratio is below the recommended threshold, illustrating that medication therapy in CF patients with mild respiratory problems is affordable in Iran.

## Discussion

How resources are allocated to health services and health care has a great impact on the level of household expenditure, hence health systems should minimize the extent of on-demand payments (out-of-pocket expenditure) which patients and their families have to spend on health care activities [34]. Numerous studies have aimed to assess the affordability of medicines in non-communicable diseases, rare diseases, essential medicines, etc. based on the WHO/HAI methodology [36]. This research was an attempt to evaluate the affordability of CRD medicines by the LPGW approach. Previous studies have mostly used the monotherapy approach to assess the affordability of CRD medicines [37], and studies gauging the affordability of these medicines in the combination therapy approach are scant. According to the study carried out by kotwani (2009) in India, the LPGW has to pay 2 days of his wages (approximately US$7) to procure a standard treatment regimen for asthma. According to this study, purchasing an inhaler of salbutamol and beclomethasone costs between 1.6 and 2.3 days’ wages for the LPGW, respectively [24].

The results of a study focusing on the availability, cost, and affordability of asthma and COPD medications in the Gambia in 2020 indicate that the costs of ICS (inhaled corticosteroid) maintenance inhalers at private pharmacies are about 15-, 26-, and 28-days’ wages for beclomethasone 50 mcg, fluticasone propionate 125 mcg, and budesonide 100 mcg, respectively. The above-mentioned study further reported that the combination of ICS/LABA (long-acting beta2-agonist) inhaler and tiotropium bromide 18 mcg costs 26 days’ and 95 days’ wages respectively for LPGW [38].

Meanwhile, the affordability assessment of CRD medicines in Iran indicated the number of days’ wages of LPGW for all generic medicines of category R were less than one day, thanks to low price and insurance coverage, and considered affordable. The findings of this study demonstrate that branded medicines included budesonide/formoterol (Symbicort®m) budesonide (Pulmicort Respules®), salmeterol/fluticasone (Flusalmex®), (Seretide®) (Fluticort Plus®), (Seroflo®) and (Salmeflo®) costs between 1.2 and 2.5 days’ wage of LPGW considered unaffordable despite 70% insurance coverage. In other words, OOP for 30% cost of these branded medicines is unaffordable for LPGW. This illustrates that insurance coverage does not necessarily guarantee patients’ treatment without undue hardship. Thus, improving patients’ financial protection needs further consideration.

According to The Global Asthma Report 2011, annual consumptions of salbutamol, beclomethasone, and budesonide purchased at private pharmacies in low and middle-income countries (LMICs) are respectively estimated to be equal to 32, 80 and 800 days’ wage [39]. Besides, median price ratio comparing medication costs to international reference prices is often higher in LMICs, making essential medications unaffordable for many patients. Therefore, addressing the affordability of CRD medications in LMICs is crucial to ensure equitable access to essential medications and improve health outcomes for patients with CRDs [40].

The affordability assessment is more complicated in combination therapy. Indeed, some of the affordable medicines in monotherapy would be unaffordable when they are used in combination with other CRD medicines, therefore, complicated patients, who need a mixed regimen, would suffer from the financial burden of their medicines.

The affordability assessment of CRD medicines in Iran as a LMIC indicated that all generic medicines of category R were affordable thanks to low price and insurance coverage. The findings of this study demonstrate that all doses of some branded medicines such as budesonide/formoterol (Symbicort®), budesonide (Pulmicort Respules®), and salmeterol/fluticasone (Flusalmex®) (Seretide®) (Fluticort Plus®) (Seroflo®) (Salmeflo®) remained unaffordable despite 70% insurance coverage. This illustrates that insurance coverage does not necessarily guarantee patients’ treatment without undue hardship. Thus, improving patients’ financial protection needs further considerations.

The affordability ratio of CRD medicines based on the combination therapy show that all medicines used in asthma, COPD, and CF are affordable except omalizumab (inj), which is unaffordable due to the high price and no insurance coverage.

The unaffordability of CRD medicines can have significant consequences for patients and healthcare systems. These consequences include limited access to necessary medications, poor disease control, excess mortality and morbidity, scarce healthcare workers, limited patient and healthcare provider training, irregular supply, catastrophic health spending, and poor healthcare efficiency. Generally, this study suggests some policy recommendations based on the Global Asthma Report 2018 to improve the affordability of CRD medications particularly in LMICs where the affordability and accessibility of essential CRD medications remain significant concerns. Policies aimed at decreasing the cost of essential CRD medications and increasing their availability and accessibility are needed to ensure that patients can receive the care and support they need to manage their condition effectively. The report also emphasizes the importance of developing and implementing national asthma management plans that are tailored to the local context and address the specific needs of patients with asthma, enhancing public awareness and education about CRD, and encouraging research and innovation in the prevention, diagnosis, and treatment of CRD [41].

### Limitation of the study

The findings of this study represent the affordability of CRD medicines in Iran, not all developing countries. Furthermore, the country-specific indirect costs will likely give more accurate results but they were not used in this study. The patient’s OOP is different in the public and private sectors but only the public sector is considered in this study. We have only investigated the affordability of CRD medicines in category R based on ATC code, while CRD patients may need multiple medications from different ATC categories. For example, a combination of antibiotics, gene modulators, etc. is needed for the treatment of CF. Hence, CF combination therapy for patients with serious and multiple complication was not investigated in this study. Based on our methodology, we have calculated the OOP of each drug by using DDD and after deducting the share of insurance coverage.

## Conclusion

In the studies conducted in this field in the world, no distinction has been made between insured and uninsured, as well as branded and generic medicines, but in our study, this distinction has been made for a proper comparison of the affordability of drugs. Also, our study was the first attempt in a developing country to assess the affordability of medicines used in asthma, COPD, and CF based on LPGW in the combination therapy treatment approach. We have calculated the affordability of medicines in the therapeutic regimen by using the affordability ratio. The findings answer the aim of the study and tried to show the health policymakers that further considerations are required to protect vulnerable households through health coverage.

## Data Availability

The datasets used and analyzed during the current study available from the corresponding author on reasonable request.

## Abbreviations

COPD: Chronic obstructive pulmonary disease
CF: Cystic fibrosis
CRDs: Chronic respiratory diseases
WHO: World Health Organization
HAI: Health Action International
LPGW: Lowest-paid unskilled government worker
DALYs: Disability-adjusted life years
GBD: Global burden of disease
IHME: Institute for health metrics and evaluation
SDI: Socio-demographic index
YLL: Years of life lost
GDP: Gross domestic product
OOP: Out-of-pocket
CHEs: Catastrophic health expenditures
IDL: Iran drug list
ATC: Anatomical therapeutic chemical
GOLD: Global Initiative for Chronic Obstructive Lung Disease
DDD: Defined daily dose
IHIO: Iran Health Insurance Organization
SSO: Social Security Organization
CFF: Cystic Fibrosis Foundation
LMICs: Low and middle-income countries
